# Supplementary material for: Acquired olfactory loss alters functional connectivity and morphology
Source: Sci Rep. 2021 Aug 12;11:16422. doi: 10.1038/s41598-021-95968-7 (PMC8361122; doi:10.1038/s41598-021-95968-7)
Supplement: Supplementary file 1 — Supplementary Information. [file 41598_2021_95968_MOESM1_ESM.pdf]

ACQUIRED OLFACTORY LOSS ALTERS FUNCTIONAL CONNECTIVITY AND MORPHOLOGY

Behzad Iravani, Moa G. Peter, Artin Arshamian, Mats J. Olsson, Thomas Hummel, Hagen H. Kitzler, &
Johan N. Lundström

SUPPLEMENTARY MATERIAL

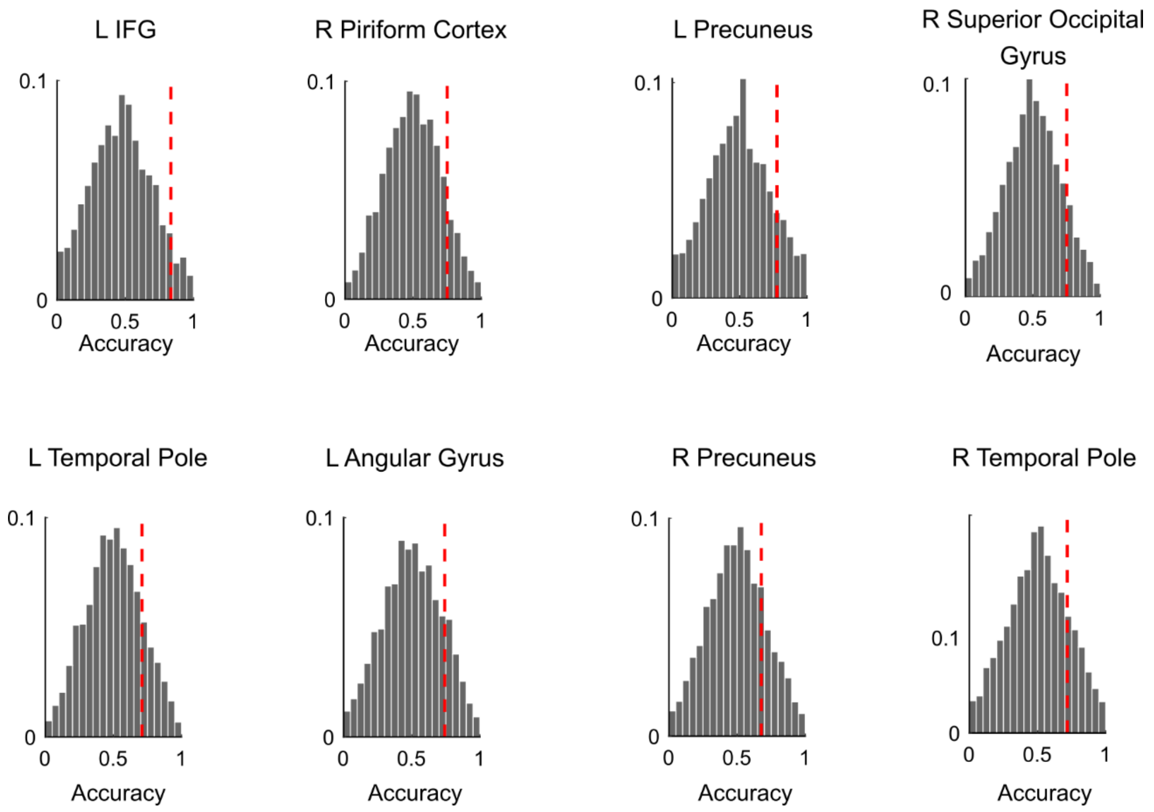

Figure S1. Permutation test of SVM. 5000 permutations performed on the clusters identified by 10-fold cross validation. The distributions show the accuracies of permuted data and red dashed line shows the observed accuracy of the original data.

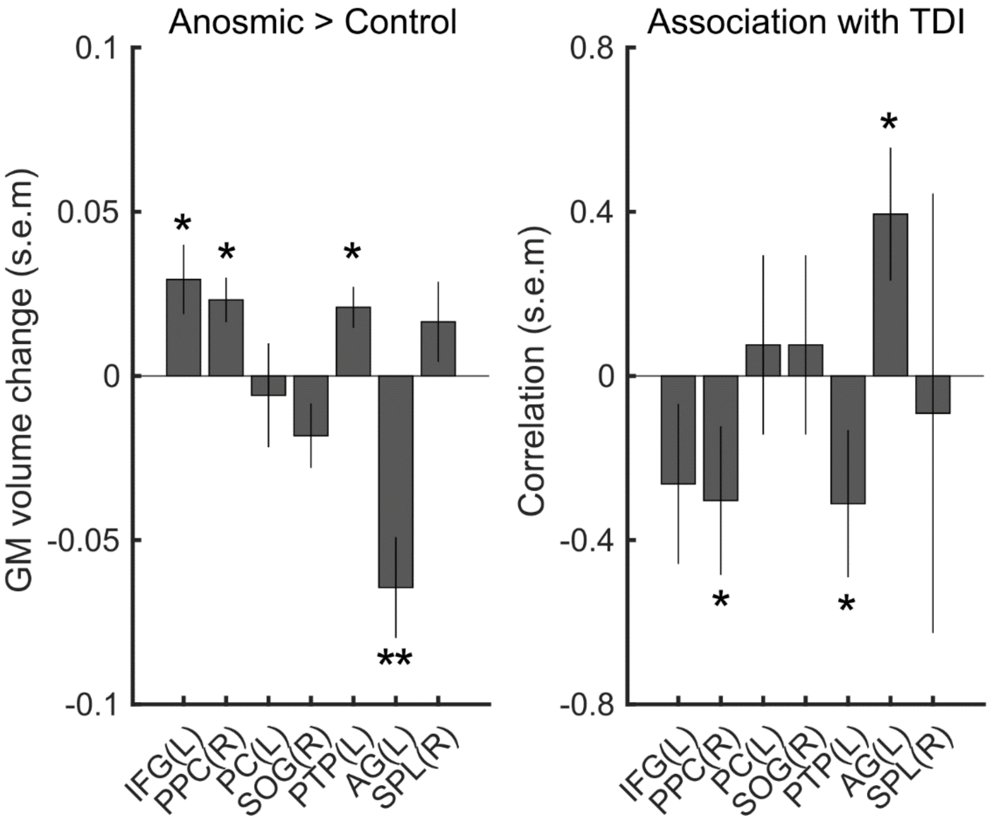

Figure S2. **Grey matte volume and its association with TDI. A**) Bar graphs represent the average grey matter (GM) volume for the seven clusters where SVM was able to classify individuals above chance level. **B**) Bar graphs show the association between GM volume of seven cluster where the SVM accuracy was above chance and TDI score. Error bars represent standard error of mean (s.e.m), * denotes $p < .05$ and ** marks $p < .005$.
